# Neuroimaging, clinical and life course correlates of normal-appearing white matter integrity in 70-year-olds

**DOI:** 10.1093/braincomms/fcad225

**Published:** 2023-08-18

**Authors:** Sarah-Naomi James, Emily N Manning, Mathew Storey, Jennifer M Nicholas, William Coath, Sarah E Keuss, David M Cash, Christopher A Lane, Thomas Parker, Ashvini Keshavan, Sarah M Buchanan, Aaron Wagen, Mathew Harris, Ian Malone, Kirsty Lu, Louisa P Needham, Rebecca Street, David Thomas, John Dickson, Heidi Murray-Smith, Andrew Wong, Tamar Freiberger, Sebastian J Crutch, Nick C Fox, Marcus Richards, Frederik Barkhof, Carole H Sudre, Josephine Barnes, Jonathan M Schott

**Affiliations:** MRC Unit for Lifelong Health and Ageing at UCL, Institute of Cardiovascular Science, University College London, London, UK; Dementia Research Centre, UCL Queen Square Institute of Neurology, University College London, London, UK; Dementia Research Centre, UCL Queen Square Institute of Neurology, University College London, London, UK; Dementia Research Centre, UCL Queen Square Institute of Neurology, University College London, London, UK; Dementia Research Centre, UCL Queen Square Institute of Neurology, University College London, London, UK; Department of Medical Statistics, London School of Hygiene and Tropical Medicine, London, UK; Dementia Research Centre, UCL Queen Square Institute of Neurology, University College London, London, UK; Dementia Research Centre, UCL Queen Square Institute of Neurology, University College London, London, UK; Dementia Research Centre, UCL Queen Square Institute of Neurology, University College London, London, UK; Dementia Research Centre, UCL Queen Square Institute of Neurology, University College London, London, UK; Dementia Research Centre, UCL Queen Square Institute of Neurology, University College London, London, UK; Dementia Research Centre, UCL Queen Square Institute of Neurology, University College London, London, UK; Dementia Research Centre, UCL Queen Square Institute of Neurology, University College London, London, UK; Dementia Research Centre, UCL Queen Square Institute of Neurology, University College London, London, UK; Centre for Medical Image Computing, University College London, London, UK; Dementia Research Centre, UCL Queen Square Institute of Neurology, University College London, London, UK; Dementia Research Centre, UCL Queen Square Institute of Neurology, University College London, London, UK; Dementia Research Centre, UCL Queen Square Institute of Neurology, University College London, London, UK; MRC Unit for Lifelong Health and Ageing at UCL, Institute of Cardiovascular Science, University College London, London, UK; Dementia Research Centre, UCL Queen Square Institute of Neurology, University College London, London, UK; Neuroradiological Academic Unit, Department of Brain Repair and Rehabilitation, UCL Queen Square Institute of Neurology, London, UK; Institute of Nuclear Medicine, University College London Hospitals Foundation Trust, London, UK; Dementia Research Centre, UCL Queen Square Institute of Neurology, University College London, London, UK; MRC Unit for Lifelong Health and Ageing at UCL, Institute of Cardiovascular Science, University College London, London, UK; Dementia Research Centre, UCL Queen Square Institute of Neurology, University College London, London, UK; Dementia Research Centre, UCL Queen Square Institute of Neurology, University College London, London, UK; Dementia Research Centre, UCL Queen Square Institute of Neurology, University College London, London, UK; MRC Unit for Lifelong Health and Ageing at UCL, Institute of Cardiovascular Science, University College London, London, UK; Centre for Medical Image Computing, University College London, London, UK; Department of Radiology and Nuclear Medicine, Amsterdam UMC, Vrije Universiteit, Amsterdam, The Netherlands; MRC Unit for Lifelong Health and Ageing at UCL, Institute of Cardiovascular Science, University College London, London, UK; Centre for Medical Image Computing, University College London, London, UK; School of Biomedical Engineering, King’s College, London, UK; Dementia Research Centre, UCL Queen Square Institute of Neurology, University College London, London, UK; MRC Unit for Lifelong Health and Ageing at UCL, Institute of Cardiovascular Science, University College London, London, UK; Dementia Research Centre, UCL Queen Square Institute of Neurology, University College London, London, UK

**Keywords:** normal-appearing white matter, microstructural integrity, brain health, vascular risk, diffusion

## Abstract

We investigate associations between normal-appearing white matter microstructural integrity in cognitively normal ∼70-year-olds and concurrently measured brain health and cognition, demographics, genetics and life course cardiovascular health. Participants born in the same week in March 1946 (British 1946 birth cohort) underwent PET-MRI around age 70. Mean standardized normal-appearing white matter integrity metrics (fractional anisotropy, mean diffusivity, neurite density index and orientation dispersion index) were derived from diffusion MRI. Linear regression was used to test associations between normal-appearing white matter metrics and (i) concurrent measures, including whole brain volume, white matter hyperintensity volume, PET amyloid and cognition; (ii) the influence of demographic and genetic predictors, including sex, childhood cognition, education, socio-economic position and genetic risk for Alzheimer’s disease (*APOE-ɛ4*); (iii) systolic and diastolic blood pressure and cardiovascular health (Framingham Heart Study Cardiovascular Risk Score) across adulthood. Sex interactions were tested. Statistical significance included false discovery rate correction (5%). Three hundred and sixty-two participants met inclusion criteria (mean age 70, 49% female). Higher white matter hyperintensity volume was associated with lower fractional anisotropy [*b* = −0.09 (95% confidence interval: −0.11, −0.06), *P* < 0.01], neurite density index [*b* = −0.17 (−0.22, −0.12), *P* < 0.01] and higher mean diffusivity [*b* = 0.14 (−0.10, −0.17), *P* < 0.01]; amyloid (in men) was associated with lower fractional anisotropy [*b* = −0.04 (−0.08, −0.01), *P* = 0.03)] and higher mean diffusivity [*b* = 0.06 (0.01, 0.11), *P* = 0.02]. Framingham Heart Study Cardiovascular Risk Score in later-life (age 69) was associated with normal-appearing white matter {lower fractional anisotropy [*b* = −0.06 (−0.09, −0.02) *P* < 0.01], neurite density index [*b* = −0.10 (−0.17, −0.03), *P* < 0.01] and higher mean diffusivity [*b* = 0.09 (0.04, 0.14), *P* < 0.01]}. Significant sex interactions (*P* < 0.05) emerged for midlife cardiovascular health (age 53) and normal-appearing white matter at 70: marginal effect plots demonstrated, in women only, normal-appearing white matter was associated with higher midlife Framingham Heart Study Cardiovascular Risk Score (lower fractional anisotropy and neurite density index), midlife systolic (lower fractional anisotropy, neurite density index and higher mean diffusivity) and diastolic (lower fractional anisotropy and neurite density index) blood pressure and greater blood pressure change between 43 and 53 years (lower fractional anisotropy and neurite density index), independently of white matter hyperintensity volume. In summary, poorer normal-appearing white matter microstructural integrity in ∼70-year-olds was associated with measures of cerebral small vessel disease, amyloid (in males) and later-life cardiovascular health, demonstrating how normal-appearing white matter can provide additional information to overt white matter disease. Our findings further show that greater ‘midlife’ cardiovascular risk and higher blood pressure were associated with poorer normal-appearing white matter microstructural integrity in females only, suggesting that women’s brains may be more susceptible to the effects of midlife blood pressure and cardiovascular health.

## Teaser

James *et al*. found normal-appearing white matter microstructural integrity in cognitive normal ∼70-year-olds was associated with concurrent brain health (cerebral small vessel disease and amyloid in men) and midlife cardiovascular health in women. This demonstrates normal-appearing white matter is an informative marker of brain health.

## Graphical Abstract

**Graphical Abstract:**
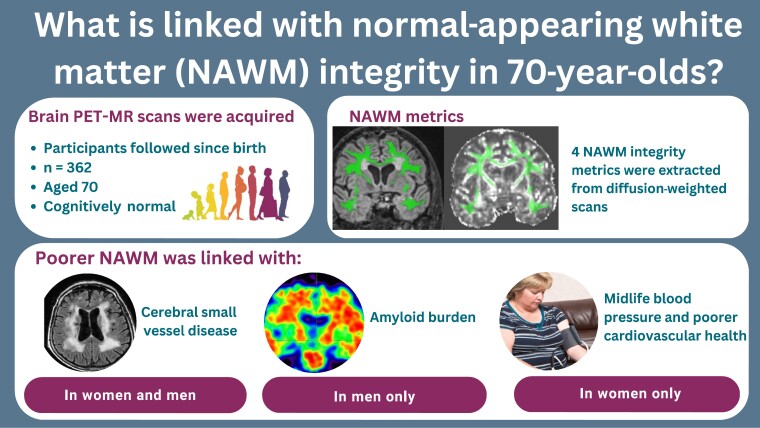

## Introduction

Associations between measures of presumed small vessel disease, e.g. white matter hyperintensities (WMHs) on fluid-attenuated inversion recovery (FLAIR) or T_2_-weighted MRI sequences, and ageing, cognitive decline and dementia have been well documented.^1-3^ Yet impaired microstructural integrity in seemingly ‘normal-appearing white matter’ (NAWM) has also been linked with ageing,^4^ cognitive decline,^5,6^ conversion from NAWM to WMH^7,8^ and exposure to vascular risk factors.^9^ Little is currently known about relationships between other disease-related imaging markers, demographic and vascular health across the life course, and NAWM integrity in later life.

NAWM integrity can be assessed using diffusion tensor imaging (DTI). Parameters typically include fractional anisotropy (FA), a measure of fibre tract directionality, and mean diffusivity (MD), a measure of the magnitude of diffusion. Reduced microstructural integrity in WM generally results in a decrease in FA and an increase in MD.^10^ Multicompartmental modelling techniques have been developed to address limitations in conventional measures, such as partial volume effects and more complex fibre organization. One technique, neurite orientation dispersion and density imaging (NODDI), can be used to derive the neurite density index (NDI), the fraction of tissue comprised of axons and dendrites, and the orientation dispersion index (ODI), a measure of the variability of neurite orientation.^11^ A lower NDI reflects less densely packed neurites (reduced microstructural integrity), and a higher ODI represents increased fanning of tracts.

The National Survey of Health and Development (NSHD, British 19246 birth cohort) is a UK population–based sample that has followed participants born in the same week in 1946 throughout their lives and amassed a wealth of prospectively collected longitudinal life course data including demographic, genetic and vascular health metrics.^12^ As part of a neuroimaging sub-study, Insight 46, 471 NSHD participants age ∼70 undertook extensive neuroimaging and clinical phenotyping including amyloid PET, structural MRI and diffusion-weighted MRI (DTI and NODDI).^13^

Drawing from this unique sample, we aimed to characterize (i) how measures of NAWM integrity correlate with concurrent brain health measures, including whole brain volume (WBV) and WMH volume (WMHV), PET amyloid burden and cognition; (ii) the influence of demographic, life course and genetic predictors, including sex, age at scan, childhood cognition, educational attainment and parental socio-economic position (SEP), and genetic risk for Alzheimer’s disease (*APOE-ɛ4*) on NAWM; and (iii) the relationship between vascular health across adulthood [vascular risk and blood pressure (BP)] and NAWM and to investigate whether these associations are modified by sex and independent of WMHV and *APOE-ɛ4*. In this population-based sample of 70 years old, we hypothesize that there will be detectable patterns reflecting poorer microstructural integrity in overtly observed NAWM. We further hypothesize that measures of reduced microstructural integrity will be linked with worse concurrent brain health measures including greater WMHV; demographics indexing disadvantaged circumstances; and worse cardiovascular health across adulthood.

## Materials and methods

Study participants were from Insight 46, a sub-study of the NSHD that initially comprised 5362 individuals born throughout mainland Britain in 1 week in March 1946. Eligibility criteria and an overview of recruitment for Insight 46^14^ are outlined in detail elsewhere. Briefly, 502 participants age 69–71 were assessed with detailed and consistent clinical, cognitive and brain imaging protocols (doi: 10.5522/NSHD/Q103). Ethical approval for Insight 46 was granted by the National Research Ethics Service (NRES) Committee London (14/LO/1173). All participants gave written informed consent.

### Imaging acquisition and processing and cognitive data at age 69–71

Simultaneous acquisition of dynamic PET and MR data was acquired at age 69–71 using a single Biograph mMR 3T PET-MRI scanner (Siemens Healthcare, Erlangen), including volumetric (1.1 mm isotropic) T_1_-weighted, T_2_-weighted and FLAIR sequences.^13^ All T_1_-weighted, T_2_-weighted, and FLAIR sequences were reviewed by a consultant neuroradiologist, and incidental findings were reported as previously described.^13^ Structural images (T_1_, T_2_, and FLAIR) were corrected for gradient non-linearity and low-frequency intensity non-uniformity with N4-bias correction.^15^ Multi-shell diffusion MRI was acquired using a twice-refocused spin echo planar imaging (EPI) sequence with two non-zero *b*-values (700 and 2000 s/mm^2^), multiple directions (12, 32 and 64 directions for the *b* = 0, 700 and 2000 s/mm^2^ scans, respectively) and an isotropic 2.5 × 2.5 × 2.5 mm resolution, with 58 slices to ensure whole brain coverage.^16^ B_0_ field maps were acquired for distortion correction of the diffusion MRI images. PET data were assessed over 10 min, ∼50 min after injection with MBq florbetapir F18 (Amyvid).

Volumetric T_1_-weighted, T_2_-weighted and FLAIR images underwent visual quality control (QC) before being processed using automated pipelines.^13^ Multi-Atlas Propagation and Segmentation^17^ were used to generate whole-brain segmentations. Total intracranial volume (TIV) was calculated using Statistical Parametric Mapping 12 (https://www.fil.ion.ucl.ac.uk/spm/). White matter hyperintensity (WMH) masks of supratentorial structures were generated from FLAIR and T_1_-weighted images using Bayesian Model Selection, an unsupervised algorithm validated for cross-sectional segmentation of WMH.^17^ Whole-brain and WMH masks were visually checked and edited where necessary.^17^

Global standardized uptake value ratios (SUVRs) were calculated from a cortical composite region of interest, normalized to the cerebellum with partial volume correction applied. Aβ positivity status was determined using Gaussian mixture modelling with two Gaussians, taking the 99th percentile of the lower distribution as the cut point (1.031, equivalent to 11.8 centiloids),^18^ whereby Aβ+ indicates greater Aβ load. Volume-weighted mean SUVR was extracted from a Geodesic Information Flows software composite cortical target region closely matched to FreeSurfer regions used in Landau *et al*.^19^ The composite consists of lateral and medial frontal, anterior and posterior cingulate, lateral parietal, and lateral temporal regions. For partial volume correction, PET images were resampled to T_1_-space and the anatomical Geodesic Information Flows regions were used to conduct iterative Yang partial volume correction with a 6.8 mm^3^ kernel optimized for the PET/MR scanner that the data were acquired on Hutton *et al*.^20^

### NAWM metrics

A NAWM mask (non-WMHV white matter) was generated and used to sample microstructural integrity measures including FA, NDI, MD and ODI.

**Figure 1:**
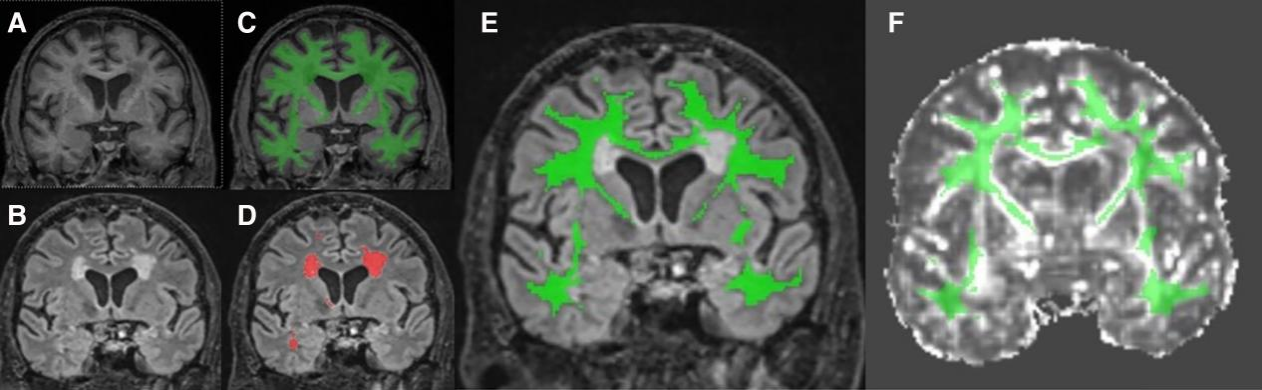
**Example of tissue segmentation.** T_1_-weighted (**A**) and FLAIR (**B**) images were segmented using automated algorithms to create white matter (WM) (**C**) and WMH (**D**) masks. The WMH mask was subtracted from the WM mask and eroded by 1 voxel to create the NAWM mask (**E**). The NAWM mask was overlayed on the FA map in the T_1_ space (**F**).

White matter masks were automatically generated from the T_1_-weighted scans using Geodesic Information Flows (GIF) software.^21^ Participant-specific masks representing NAWM were generated by subtracting the Bayesian Model Selection WMH masks from the GIF white matter masks using NiftySeg (https://github.com/KCL-BMEIS/NiftySeg), before being eroded by 1 voxel (see Fig. 1). Diffusion-weighted images were corrected for inter-volume motion using linear registration and eddy currents using FSL’s Eddy tool (https://fsl.fmrib.ox.ac.uk/fsl/fslwiki/eddy), followed by correction for EPI susceptibility distortion using field maps, with volume-preserving modulation based on the Jacobian determinants. Diffusion tensor models were fitted to b700 and b2000 diffusion shells using NiftyFit.^22^ The NODDI model was then fitted to the combined shells using the NODDI toolbox (http://mig.cs.ucl.ac.uk/index.php?n=Tutorial.NODDImatlab). All images underwent visual QC to assess acquisition, pre- and post-processing motion, coverage, blurring, image wrap-around and contrast problems, adequate CSF suppression on FLAIR imaging, segmentation or processing artefacts, sufficient correction of geometric distortion, and slice-wise signal dropout on diffusion MRI (using correlation plots between adjacent slices). Images failing the QC process for DTI were removed before running the analysis for FA or MD metrics. NODDI models required an extra semi-automated QC step, and images failing this QC were removed before running analyses including NDI or ODI.

Given that NAWM metrics vary greatly across the brain, averaging absolute values of NAWM metrics over such large regions are not intuitively meaningful. We employ a similar approach used in PET imaging^23^ to ease interpretation of abnormality of the NAWM metrics. To do so, we derived a model of ‘healthy’ NAWM metrics (FA, MD, NDI, and ODI) from a subset of participants with a very limited amount of WMH (<1 mL) and compared each participant’s divergence from these models by calculating voxel-wise *z*-scores per diffusion map. For the diffusion metric of interest, the *z*-score at each voxel therefore represents how much that voxel deviates from what is assumed to be ‘healthy’ to help ease interpretation of abnormality. This approach also enables us to allow for comparisons across the different metrics. The mean *z*-score over the individual’s NAWM mask was then calculated for each diffusion metric. These mean standardized diffusion metrics were used as the outcome measures in the regression analyses.

### Cognitive function at age 69–71

The Preclinical Alzheimer Cognitive Composite (PACC) was used as the main cognitive outcome at age 69–71.^24^ This composite consists of the Mini-Mental State Examination; logical memory delayed score from the Wechsler Memory Scale-Revised, a test of verbal episodic recall; digit-symbol substitution test from the Wechsler Adult Intelligence Scale-Revised, a test of executive function and psychomotor speed; and the 12-item Face-Name test, a measure of free memory recall.

### Life course predictors

#### Demographic predictors of sex, educational attainment, parental SEP and childhood cognition, APOE-ɛ4 carrier status

Sex was ascertained at birth. Childhood cognitive ability was measured at age 8 using four tests of verbal and non-verbal ability devised by the National Foundation for Education Research. The sum of scores from these four tests was standardized into a *z*-score representing overall cognitive ability, standardized to the full cohort. The highest educational attainment achieved by 26 years was grouped as no qualifications, education up to age 16 (O-levels or equivalent) and education from age 17 onwards (A-levels or higher). Parental SEP was derived from paternal occupational class and coded according to the UK Registrar General’s Standard Occupational Classification as manual and non-manual. Genotyping of the two single-nucleotide polymorphisms, rs439358 and rs7412, was used to determine *APOE-*ɛ4 genotype and categorized as ɛ4 carriers and non-carriers.

#### Cardiovascular predictors across the life course including FHS-CVS and BP

Framingham Heart Study Cardiovascular Risk Scores (FHS-CVSs) were derived from measurements collected on home visits by research nurses when participants were ages 36 (early adulthood), 53 (midlife) and 69 (early late life), prior to their Insight 46 visit. The FHS-CVS provides a 10-year risk of cardiovascular events from a weighted sum of age, sex, systolic BP (SBP), antihypertensive medication usage (yes/no), history of diabetes (yes/no), current smoking (yes/no) and body mass index (calculated as weight in kilograms divided by height in meters squared).^25^ Smoking status was defined by questionnaire at ages 36, 53 and 69. Diabetes mellitus status was based on self-reported diagnosis at age 36, and at ages 53 and 69, it was based on self-reported diagnosis or a haemoglobin A_1C_ level of 6.5% or more. Body mass index was measured at ages 36, 53 and 69 by research nurses.

Seated BP was measured in the upper arm twice after 5 min of rest across adulthood at ages 36, 43, 53, 60–64 and 69. In early adulthood (36 and 43 years), a Hawksley random zero sphygmomanometer was used, and from midlife onwards (53, 60–64 and 69 years), an Omron HEM-705 automated digital oscillometric sphygmomanometer was used. To ensure compatibility, published conversion equations were applied.^26^ The second BP measure was used for analyses (unless only one measure was available). SBP and diastolic BP (DBP) change for the periods between early adulthood (ages 36 and 43), midlife (ages 43 and 53) (ages 53 and 60–64) and early late life (ages 60–64 and 69), conditional on earlier measurements, was calculated as the residual from the regression of each BP measure (from 43 years of age) on the earlier measures for each sex, using individuals with available data at all time points.^26^ Residuals represent changes in BP that differed from changes expected on average given the earlier BP. Residuals were standardized, allowing comparison between periods.

### Statistical analysis

**Figure 2:**
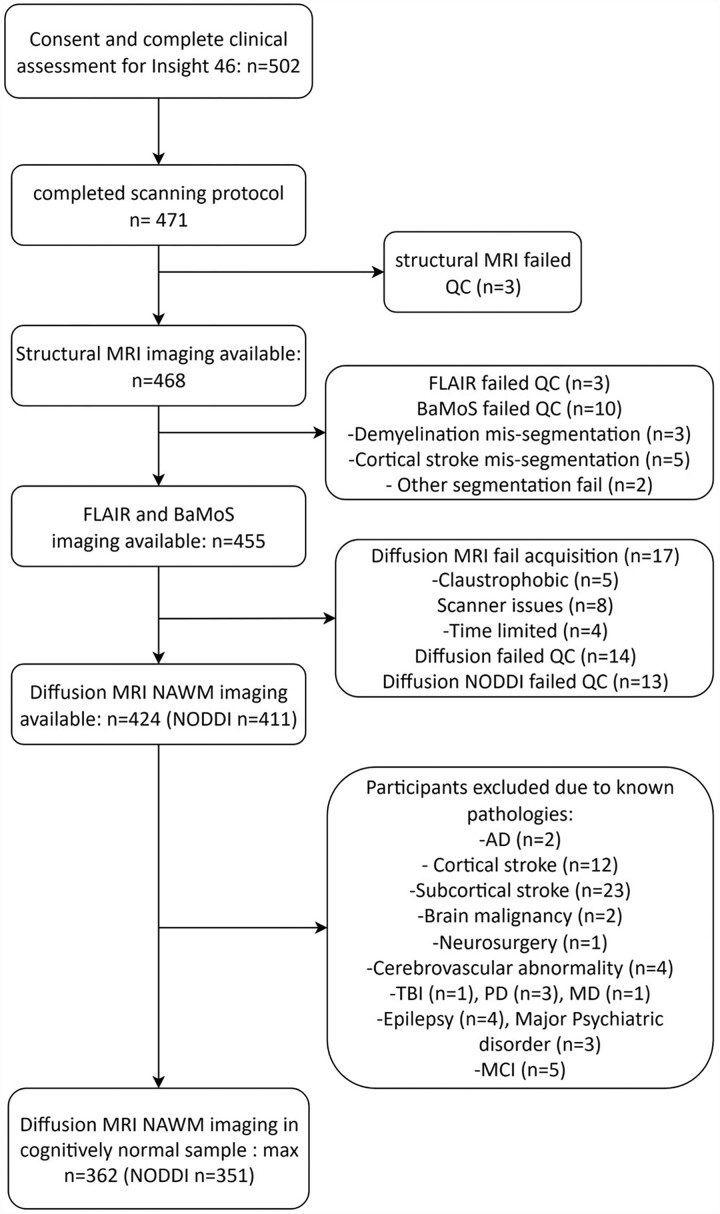
**Flowchart of participant inclusion criteria.** BaMoS = Bayesian Model Selection; AD = Alzheimer’s disease; TBI = traumatic brain injury; PD = Parkinson’s disease; MCI = mild cognitive impairment.

We used Stata 17.0 (StataCorp) for all statistical analyses. Only participants with good quality T_1_-weighted, FLAIR and diffusion MRI scans were included. Ten participants whose Bayesian Model Selection segmentation failed QC were also excluded. Participants who were cognitively impaired or had a neurological or major psychiatric condition were excluded from analyses. See Fig. 2 for a flowchart of participant exclusions.^27,28^

#### Multiple testing approach

The Benjamini and Hochberg step-up procedure was applied to control for the false discovery rate (FDR) set at 5%.

### Concurrent pathological correlates at age 69–71

Linear regression models were used to analyse the association between WBV (continuous), log-transformed WMHV (continuous) and amyloid load (dichotomous) with each standardized NAWM diffusion metrics (FA, MD, NDI, and ODI). WMHV and WBV were transformed into *z*-scores (with a mean of 0 and a SD of 1). The concurrent pathology measures were assessed in the same model to estimate the independence of effects per NAWM metric. All models were adjusted for age at scan and sex. The models investigating the effect of WMHV and WBV on NAWM diffusion metrics were additionally adjusted for total intracranial volume. A differential influence of sex on the relationship between pathology and NAWM was tested using an interaction term. The marginal effects are shown (the relationship for each sex) if there was evidence of a sex interaction (*P* < 0.1).

### Concurrent cognition correlates at age 69–71

Linear regression models were applied to analyse the relationship between the PACC and each standardized NAWM diffusion metric, adjusting for age at scan and sex. Models were fitted with the four cognitive sub-tests in the same model, to estimate the independence of effects, and each NAWM metric, adjusting for age at scan and sex. A differential influence of sex on the relationship between cognition and NAWM was tested using an interaction term, and, if significant, marginal effects were reported.

### Life course predictors: demographic predictors, childhood cognition and APOE-ɛ4 carrier status

Linear regression models were conducted to characterize the relationship between demographic predictors (sex, age at scan, educational attainment and parental SEP), childhood cognition and genetic risk for Alzheimer’s disease (APOE-ɛ4 status) with each NAWM metric at 69–71 years of age. The predictors were assessed in the same model to estimate the independence of effects from these demographic variables.

### Life course predictors: cardiovascular risk and BP

Linear regression models were fitted to characterize the relationship between a range of life course cardiovascular health metrics in early, mid and later adulthood (FHS-CVS at ages 36, 53 and 69; SBP and DBP at ages 36, 43, 53, 60 and 69; and BP change variables between ages 36 and 43; 43 and 53; 53 and 60; and 60 and 69) with each NAWM metric. Measures were transformed into *z*-scores (with a mean of 0 and a SD of 1). These cardiovascular predictors were tested in separate models, but all were adjusted for age at scan and sex. A differential influence of sex on the relationship between cardiovascular health and NAWM was tested using an interaction term, and, if significant, marginal effects were reported.

### Sensitivity analyses

To investigate whether associations were independent of the amount of presumed cerebral small vessel disease present, we additionally re-ran the significant models adjusting for WMHV and total intracranial volume. To investigate whether associations were independent of APOE-ɛ4 status, we additionally re-ran the significant models adjusting for APOE-ɛ4 status.

An overview table of the analytical approach to covariates, interactions and sensitivity analyses is outlined in Supplementary Table 1.

## Results

Participant characteristics are shown in Table 1 for participants with available data and excluding those with any neurological disorder.

**Table 1:** Participant characteristics

	*N*	Overall	Males	Females
Sample	362		183	179
Age at scanning	362	70.6 (0.7)	70.6 (0.7)	70.7 (0.7)
Educational attainment up to age 26	362			
No qualifications		57 (16%)	27 (15%)	30 (17%)
Education up to age 16 (O-levels or equivalent)		109 (30%)	39 (21%)	70 (39%)
Education from age 17 onwards (A-levels or higher)		196 (54%)	117 (64%)	79 (44%)
Parental SEP	357			
Non-manual		203 (57%)	113 (62%)	90 (51%)
Manual		154 (43%)	69 (38%)	85 (49%)
*APOE-ɛ4* carrier	361	108 (30%)	55 (30%)	53 (30%)
FHS-CVS %:				
At age 36	322	2.6 (1.5, 3.6)	3.5 (2.9, 4.3)	1.5 (1.2,1.9)
At age 53	351	10.4 (6.3, 15.4)	14.9 (11.8,18)	6.2 (4.6, 8.5)
At age 69	352	23.3 (14.6, 34.5)	33.2 (26.4, 39.7)	14.5 (10.3,18.8)
SBP:				
At age 36	324	119.7 (13.7)	125.6 (12.7)	113.7 (12.1)
At age 43	341	124.0 (14.0)	129 (13)	118.9 (13.1)
At age 53	352	133.3 (19.4)	137.9 (19.7)	128.6 (17.9)
At age 60–64	361	134.8 (17.0)	138.3 (17.5)	131.2 (15.7)
At age 69	357	132.2 (16.2)	134.1 (15.2)	130.3 (17.1)
DBP:				
At age 36	324	78.2 (9.9)	81.2 (9.6)	75 (9.3)
At age 43	341	80.3 (8.9)	83.2 (8.4)	77.4 (8.5)
At age 53	352	83.1 (11.9)	86.6 (12.3)	79.4 (10.3)
At age 60–64	361	76.9 (9.5)	78.7 (9.9)	75.1 (8.7)
At age 69	357	73.2 (10.2)	74.1 (10.8)	72.3 (9.5)
Hypercholesterolaemia at 69 years of age	362	288 (80%)	137 (75%)	151 (85%)
Body mass index at age 70	362	27.3 (4.2)	27.6 (3.8)	27.1 (4.7)
Diabetes at age 70	358	34 (10%)	18 (10%)	16 (9%)
*Imaging and cognition at age 70*				
Amyloid positivity (Aβ+)	359	57 (16%)	25 (14%)	32 (18%)
SUVR	359	1.0 (0.2)	0.6 (0.1)	0.6 (0.1)
Global WMHV, mL	362	2.9 (1.6, 6.1)	2.7 (1.6, 5.6)	3.4 (1.7,6.8)
Whole brain volume, mL	362	1105.6 (98.6)	1158.5 (84.9)	1051.6 (80.8)
Total intracranial volume, mL	362	1434.0 (134.4)	1522.6 (105.8)	1343.5 (94.4)
PACC score	362	0.1 (0.7)	−0.1 (0.7)	0.2 (0.6)
Digit symbol	362	48.7 (10.2)	47.4 (10.5)	50.1 (9.8)
Logical memory	362	11.6 (3.6)	10.7 (3.6)	12.5 (3.3)
Face-Name score	362	66.9 (17.1)	63.3 (17.6)	70.4 (15.9)
Mini-Mental State Examination score	362	29.3 (0.9)	29.2 (0.9)	29.4 (0.8)

Values shown are *n* (%), mean (SD) or median (q1, q3).

### Concurrent imaging correlates at age 69–71

**Figure 3:**
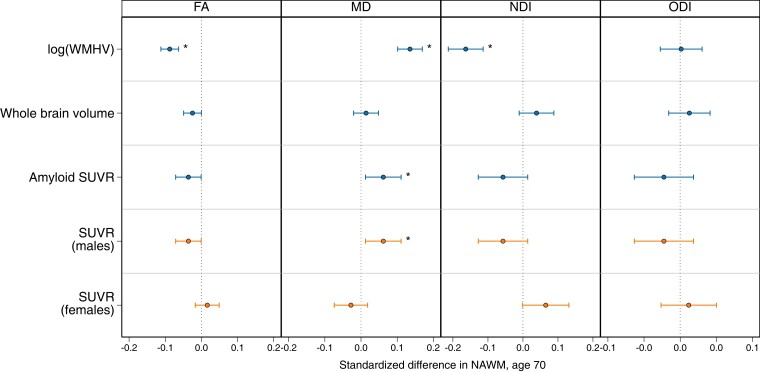
**Associations between concurrent imaging correlates with standardized global mean NAWM parameters of FA, NDI, MD and ODI at age 69–71.** Regression coefficient plot of the standardized estimates that reflect the differences in mean of the standardized NAWM outcome by 1 SD change in the predictor variable. Lines indicate the widths of the 95% confidence intervals. All concurrent imaging correlates were assessed in the same model (mutually adjusting for WMHV, brain volume and amyloid) and adjusted for sex and age at scan, per NAWM metric. A sex and amyloid SUVR significant interaction emerged, so amyloid SUVR results show the marginal effects by sex. Associations that survived FDR correction are indicated by an asterisk.

Higher WMHV and SUVR were independently and significantly associated with worse NAWM microstructural measures (both were associated with lower FA and higher MD; in addition, WMHV was also associated with lower NDI) (Fig. 3). A significant sex interaction emerged for amyloid SUVR (*P* < 0.01): higher SUVR was associated with worse NAWM microstructural metrics in males (lower FA and higher MD), but no associations between SUVR and NAWM were significant in females (Fig. 3). The association between WBV and FA did not survive false discovery rate correction. There was no evidence of sex interactions with WMHV or BV or attenuation of effects with adjustment for APOE-ɛ4 (Supplementary Table 2).

### Concurrent cognitive correlates at age 69–71

**Figure 4:**
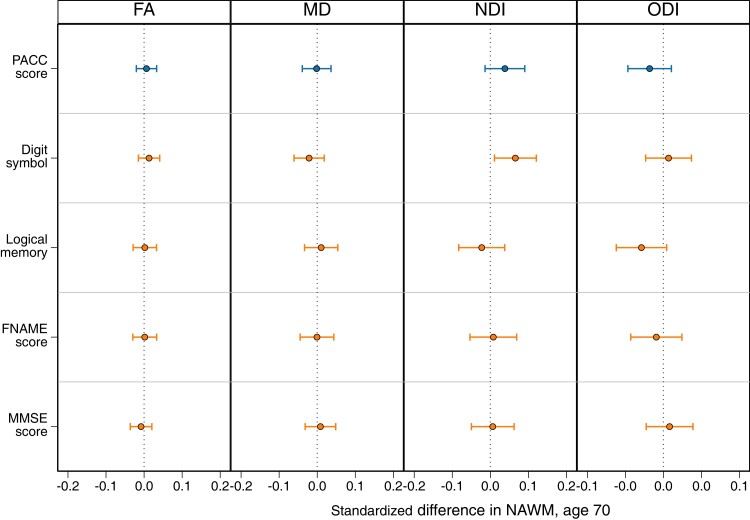
**Associations between concurrent cognition correlates (PACC and its four sub-components) with standardized global mean NAWM parameters of FA, NDI, MD and ODI at age 69–71.** Regression coefficient plot of the standardized estimates that reflect the differences in mean of the standardized NAWM outcome by 1 SD change in the predictor variable. Lines indicate the widths of the 95% confidence intervals. All models adjust for age at scan and sex; all sub-components of the PACC were assessed in the same model, per NAWM metric. Associations that survived FDR correction are indicated by an asterisk. MMSE = Mini-Mental State Examination.

Neither cognitive performance as measured using the PACC total score nor its sub-components were significantly associated with any NAWM microstructural metrics (Fig. 4).

There was an association between digit symbol and higher NDI, but this did not survive FDR correction (Supplementary material). There was no evidence of sex interactions or attenuation of effects with adjustment for WMHV or APOE-ɛ4 (Supplementary Table 3).

### Life course predictors

**Figure 5:**
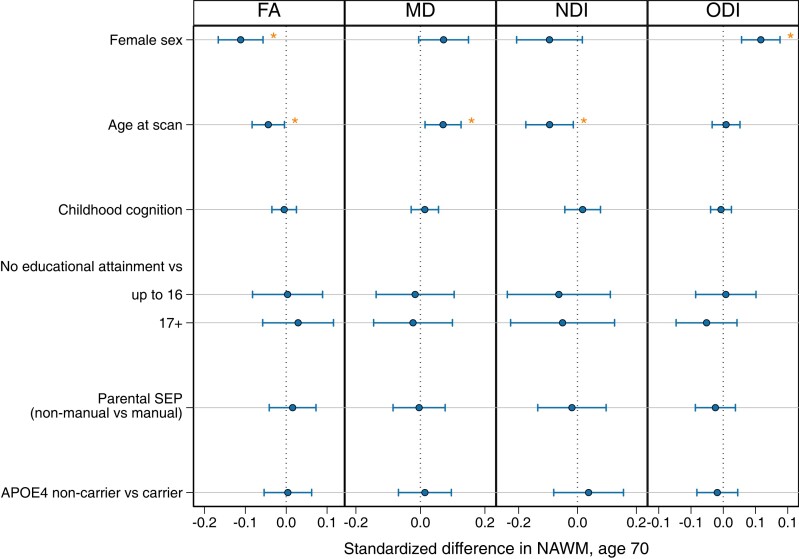
**Associations between life course demographics with mean NAWM parameters of FA, NDI, MD and ODI at age 69–71.** Regression coefficients and 95% confidence intervals of the standardized estimates reflect the differences in mean of the standardized NAWM outcome by 1 SD change in the predictor variable. Estimates for age reflect the differences in mean associated with a 1-year increase in age and for an increase of 1 SD for childhood cognition. Lines indicate the widths of the 95% confidence intervals. The predictors were assessed in the same model to estimate the independence of effects, per NAWM metric. Associations that survived FDR correction are indicated by an asterisk.

Regression models demonstrated that female sex and older age at scan were independently associated with worse NAWM microstructural measures (female sex with lower FA; older age with lower FA, NDI and higher MD) (Fig. 5). Female sex was also associated with higher ODI. Other demographic variables, including childhood cognition, education, parental SEP and APOE-ɛ4 status, were not associated with NAWM microstructural measures. There was evidence of an attenuation of all the associations between female sex and older age with NAWM when associations were adjusted for WMHV. There was no attenuation of effects with adjustment for APOE-ɛ4 status (Supplementary Table 4).

### Life course predictors: cardiovascular risk score and BP

**Figure 6:**
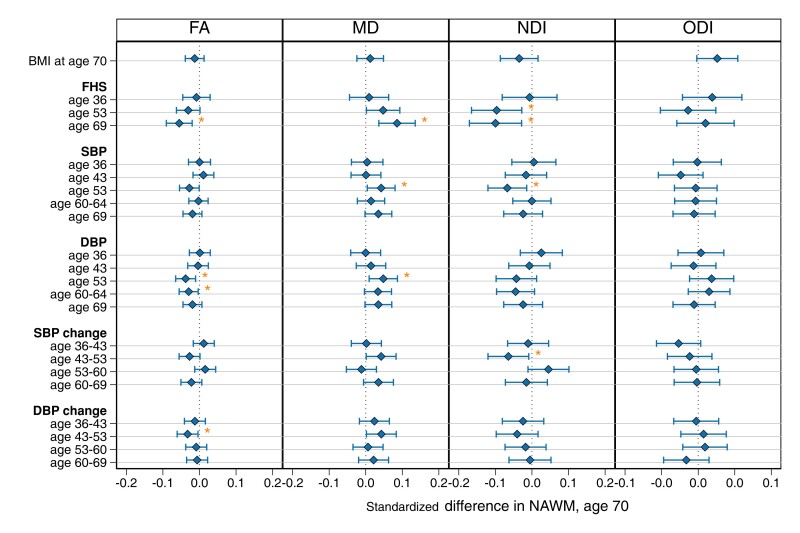
**Life course vascular risk and BP associations with mean NAWM parameters of FA, NDI, MD and ODI at age 69–71.** Regression coefficient plot of the standardized estimates that reflect the change (differences in mean) of the standardized NAWM outcome by 1 SD change in the predictor variable. Lines indicate the widths of the 95% confidence intervals. All models were run separately and adjusted for age at scan and sex and are FDR corrected. Associations that survived FDR correction are indicated by an asterisk.

Higher cardiovascular risk scores—FHS-CVS—in later adulthood (at age 69) were associated with worse NAWM metrics (lower FA, NDI and higher MD) (Fig. 6). Higher SBP and DBP in midlife (at 53) were associated with NAWM metrics (SBP with higher MD and lower NDI; DBP with higher MD and lower FA) (Fig. 6). Greater increases in SBP and DBP in midlife (between ages 43 and 53) were associated with NAWM metrics (SBP with lower NDI; DBP with lower FA) (Fig. 6). Higher DBP at age 60–64 was also associated with lower FA. All *P*-values were FDR corrected. There was no evidence of attenuation of these effects with adjustment for WMH volume or APOE (Supplementary Table 5).

### Models with sex interactions

**Figure 7:**
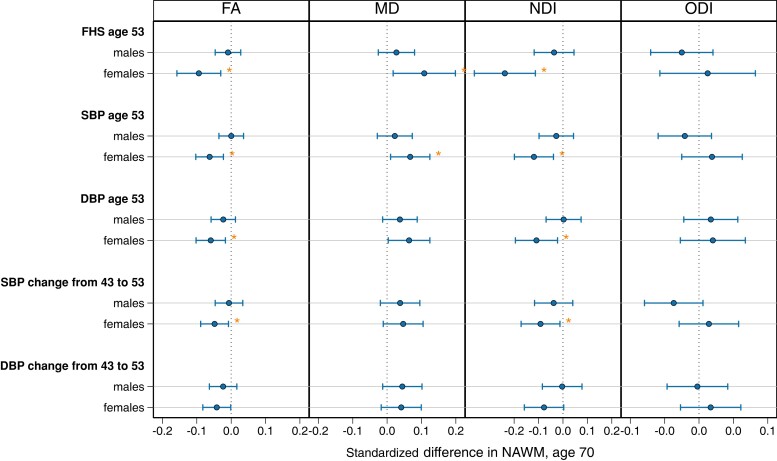
**Marginal effects demonstrating sex differences in vascular risk and BP associations at age 53 with mean NAWM parameters of FA, NDI, MD and ODI at age 69–71.** Regression coefficient plot of the standardized estimates that reflect the change (differences in mean) of the standardized NAWM outcome by 1 SD change in the predictor variable. Lines indicate the widths of the 95% confidence intervals. Models for each exposure were run in pooled analysis and adjusted for age at scan and are FDR corrected. Associations that survived FDR correction are indicated by an asterisk.

Statistically significant sex interactions emerged for cardiovascular scores and BP (SBP and DBP) in midlife (at age 53), and BP (SBP and DBP) changes in midlife (between ages 43 and 53; Supplementary Table 5). The marginal effects (slope per sex) were subsequently displayed (Fig. 7). In females, NAWM metrics at age 70 were associated with higher midlife (age 53) FHS-CVS scores (lower FA and NDI and higher MD), higher SBP (lower FA and NDI and higher MD), higher DBP at age 53 (lower FA and NDI) and greater SBP change (between age 43 and 53: lower FA and higher NDI) (Fig. 7). In males, there were no significant associations between midlife cardiovascular scores or BP with NAWM metrics.

### Adjustments

There was no evidence of attenuation of these effects with adjustment for WMH volume or APOE-ɛ4 (Supplementary Figs. 1–6). All *P*-values were FDR corrected.

## Discussion

In a population-based sample of dementia-free individuals, all born in the same week and aged ∼70 at the time of imaging, we investigated associations between NAWM integrity and concurrently measured brain pathology and demographic, cognitive, genetic and cardiovascular health measures across the life course. We found that NAWM measures reflecting poorer microstructural integrity were associated with greater WMHV, supporting the notion that altered NAWM and WMH may be part of a partially overlapping pathological process.^7,8^ Our findings also suggest that NAWM is not necessarily ‘normal’ and that WMHV does not fully capture white matter pathology.^4^ A higher amyloid load (SUVR) was associated with reduced microstructural integrity in males but not females. Being female and being scanned at an older age over the 2-year assessment period were independently associated with NAWM microstructural differences, but this was explained by differences in cerebral small vessel disease; no evidence emerged for associations between NAWM and childhood cognition, education and the biggest genetic risk factor for ‘sporadic’ Alzheimer’s disease (*APOE-ɛ4*). Overall, poorer cardiovascular health in later life was associated with impaired microstructural NAWM integrity in later life in a pooled sample. However, greater ‘midlife’ cardiovascular risk and higher BP were associated with poorer NAWM microstructural integrity in females only, suggesting the increased susceptibility of midlife BP and cardiovascular health for subsequent white matter brain health for women. Together, our findings suggest that assessment of NAWM provides important additional information to overt white matter disease. These findings support the concept that modifiable midlife cardiovascular risk factors are associated with covert late-life brain health, particularly in women.

### Concurrent pathological and cognitive correlates at age 69–71

The findings that increased WMHV was significantly associated with NAWM diffusion metrics add to a growing body of evidence that poorer white matter microstructural integrity, even in NAWM, is linked with overt white matter disease burden,^6-8,29^ and this may be an important outcome measure for understanding early white matter pathophysiology throughout adulthood.^6-8,29^ Notably, whilst WMH is fairly low in this sample, tract-based DTI in a cross-sectional community study of ∼50-year-olds similarly found that lower mean NAWM FA was associated with hyperintensity volumes;^29^ these findings suggest that these relationships can be detected even at low levels of focal white matter disease burden. Whilst we report cross-sectional data, our findings support longitudinal studies that have found lower microstructural integrity in NAWM is associated with the subsequent development of WMH.^7,8^ These studies suggest that there may be a continuous process pathway of white matter degradation including demyelination and axonal loss due to chronic ischaemic vascular processes^7,8^ and reduced cerebral blood flow.^30^ However, there is evidence that WMH can be reversible,^31^ and it is plausible that there is a dynamic process between NAWM and WMH, particularly at low levels of disease burden.^32^ NAWM DTI metrics may be important for identifying early—and potentially more reversible—vascular damage and for evaluating progression for clinical trials.

We found that a higher amyloid load (SUVR) was associated with lower FA, higher MD and lower NDI in NAWM in males but not in females. Associations between white matter microstructural integrity and amyloid load in cognitively normal participants have previously been reported.^33-35^ Although beyond the scope of this study, further investigations into the sex differences of this relationship are warranted and could reflect differences in the underlying aetiology causing NAWM microstructural alterations, such as amyloid-induced WM alterations,^34^ or due to sexual dimorphism in white matter organization.^36^

There was little evidence that concurrent cognitive performance was related to NAWM metrics at age ∼70. As our analysis only included cognitively healthy participants, further follow-up in the study, when more study members will be expected to develop cognitive changes and disease, will allow investigation of the link between NAWM metrics and cognitive impairment and decline.

### Life course predictors: demographics, childhood cognition and APOE-ɛ4 carrier status

Being female and having a later age at scan were independently associated with measures indexing differential microstructural integrity, but these findings attenuated with adjustment for WMH, a marker of cerebral small vessel disease. This suggests that the sex- and age-related alterations in NAWM we observed are partly explained by a greater burden of cerebral small vessel disease, which provides evidence that alterations in NAWM and WMH may be part of an overlapping pathological process.^7,8^ Our results are consistent with those from a previous study, in middle-aged to older adults, that found lower FA and higher ODI in females compared with males across multiple tracts.^37^ There is growing evidence that older women are more likely to have greater WMHV despite a lower prevalence of vascular risk factors,^38-40^ suggesting a higher susceptibility to white matter damage.^41^ The age range of participants scanned was very narrow, and so rather than an age effect *per se*, it is possible that the observed age effect is due to recruitment bias: those participants recruited later into the study may be less healthy and more likely to have greater WMHV than those who were keen to enrol at an early stage.

We found no associations between earlier life demographics (childhood cognition, educational attainment and parental SEP) and microstructural integrity measures in NAWM. This does not exclude the possibility that there may be regional associations, not detectable when looking at summary measures across the brain, however. A previous study, using data from the population-based Lothian birth cohort, did find that higher childhood cognition was associated with higher FA in the centrum semiovale but not in other regions of interest.^42^

### Life course predictors: cardiovascular health

Worse cardiovascular health only in later life (age 69) was associated with worse NAWM microstructural integrity across both sexes. However, worse cardiovascular health and higher BP in midlife (at age 53) and increases in BP in midlife (between ages 43 and 53), were associated with measures indexing worse NAWM microstructural integrity at age 70, in women only. These relationships were independent of presumed cerebral small vessel disease present, suggesting that NAWM provides important additional evidence to overt white matter disease of adverse brain health effects, particularly in women. This is in line with findings from a tract-based DTI cross-sectional community study of ∼50-year-olds showing that hypertension at this age was associated with lower NAWM FA, independent of adjustment for white matter burden.^29^

The sex differences we observed between midlife cardiovascular health and BP with late-life NAWM microstructural integrity are in keeping with previous studies that show a stronger association between BP and FA in middle-aged females than males.^41,43^ Stronger associations in women between raised BP and higher WMHV^41,43^ as well as between midlife hypertension and later dementia^44^ have also been found previously. This generally adds to a pattern of growing evidence suggesting, despite a lower prevalence of midlife cardiovascular risk factors, women with poorer cardiovascular health have a greater susceptibility to white matter damage.^41^

There are known sex differences in the susceptibility to cardiovascular disease. At younger ages, males have greater risk, but in older age, female risk surpasses males.^45^ Survivor bias could explain such patterns, but there is also a growing realization that cardiovascular disease is under-recognized, under-diagnosed and under-treated in females.^45^ Declining levels of protective oestrogen during menopause,^46^ or pregnancy-related issues, including gestational hypertension of pre-eclampsia,^47-50^ could increase the susceptibility to midlife cardiovascular health in women. Differences in lifestyle behaviours, such as smoking, alcohol consumption and physical activity, and differential biological mechanisms linking behavioural factors with cardiovascular health could differ by sex. Further work is warranted to understand the mechanisms of exacerbated risk of midlife cardiovascular health on later-life white matter brain health in women, expanding on the body of evidence of sex differences in cardiovascular disease. For example, studies using MRI and DTI in children and adolescents suggest there is sexual dimorphism in the structural development of white matter and microstructural organization^36^ with an implicated role of hormones and puberty.^51^ Further investigation into sex differences of the structural organization of white matter in the ageing brain is critical.

Previous analyses in this sample demonstrated that cardiovascular risk and rising BP, particularly in early midlife between ages 43 and 53, were linked with greater WMHV at age 69–71, emphasizing the importance of midlife vascular risk on subsequent white matter disease burden.^26^ The current findings expand on this body of work by demonstrating that BP changes in this period of midlife are not only linked to overt white matter disease burden ∼20 years later but also linked to NAWM microstructural integrity, independently of the presumed cerebral small vessel disease present. Together, these findings support the notion that NAWM is not necessarily ‘normal’ and that WMHV does not fully capture white matter pathology and damage.^4^ Our findings also provide evidence that midlife BP and cardiovascular health are associated with poorer white matter brain health decades later, which may not be fully captured by conventional (MR) imaging. Whilst it is not possible to directly infer the underlying microstructural changes related to diffusion metrics without supporting histology, microstructural changes may be related to axonal loss, demyelination, and gliosis.^52^ NAWM may additionally already have low perfusion or microstructural changes,^53^ but the pathophysiological changes may be more reversible than overt WMHs.^54^ Detecting differences observed in microstructural integrity in NAWM could therefore be important to characterize early subtle pathological changes linked with vascular health. Whilst longer-term follow-up is required to determine whether these changes have implications for cognitive changes in later life, this supports the importance of emphasizing the role of midlife BP management and cardiovascular health for improving later-life health.

We found no evidence that increased BP in early adulthood (36–43 years), or poorer cardiovascular health in early adulthood (age 36), was associated with worse NAWM microstructural integrity later in life. Interestingly, in our previous study in this cohort, we found no association between BP at age 36 and whole WMHV at age 70.^26^ We did find, however, that higher BP in these earlier ages was associated with smaller brain and hippocampal volume at age 70, independent of WMHV load.^26^ Together, these findings suggest that BP may be linked with brain volume and WMHV through differential pathways. For example, early adulthood BP may influence brain volume through pathways related to tau pathology,^55^ hypertension-related infarction,^56^ or shared common predictors (e.g. genetics), whereas the relationship between midlife BP and white matter damage may be mediated by changes in perfusion and inflammatory processes.^54^ Since only cognitively unimpaired participants age ∼70 were included in our study, and few individuals have hypertension in early adulthood, the impact of subtle hypertension influences in early adulthood on late-life NAWM metrics may not be apparent in this cohort.

## Strengths and limitations

This study has several strengths. We used a population-based birth cohort with data spanning 70 years, enabling prospectively ascertained demographic and adulthood vascular health. Participants were born in the same week, which reduces the risk of confounding by age. Participants were scanned around ∼70 years old, where pathology is expected to accumulate, but clinical manifestations of dementia are still limited. In this context, some of the findings reported here may reflect the relatively early stage of pathophysiological continuum of diseases that we expect some participants to be in, potentially many years before onset of Alzheimer’s disease–related neurodegeneration.

This study also has several limitations. First, the British population in Insight 46 is a cohort of selectively healthy, socially advantaged and exclusively White British participants,^14^ reducing generalizability to other populations. Second, this analysis uses imaging measures at one time point; ongoing assessments (including tau-PET) in our sample are planned to gain more detailed information about the pathological and demographic correlates with longitudinal changes in NAWM. Third, WMHV is not randomly distributed in the brain and specific tracts are more likely to be excluded from the NAWM than others, potentially introducing some bias in the NAWM diffusion metrics. Further investigations are needed to understand if the sex differences observed are due to true microstructural differences rather than potential bias induced by WMH distribution/load differences. Future work will also address whether medications, such as statins and ACE inhibitors, affect these relationships.

In summary, poorer cardiovascular health and higher BP in midlife and increases in BP in early midlife were associated with poorer NAWM microstructural integrity measures decades later in cognitively unimpaired participants in women. Poorer cardiovascular health in late life was also associated with poorer NAWM microstructural integrity measures. These relationships were not fully explained by WMHV, suggesting that assessment of NAWM provides important additional information to overt white matter disease. These findings support the concept that modifiable midlife cardiovascular risk factors are associated with covert late-life brain health, particularly in women.

## Supplementary material

Supplementary material is available at *Brain Communications* online.

## Supplementary Material

fcad225_Supplementary_Data

## Data Availability

Anonymized data will be shared by request from qualified investigators (skylark.ucl.ac.uk/NSHD/doku.php).
