# Supplementary material for: Neuroimaging, clinical and life course correlates of normal-appearing white matter integrity in 70-year-olds
Source: Brain Commun. 2023 Aug 18;5(5):fcad225. doi: 10.1093/braincomms/fcad225 (PMC10481255; doi:10.1093/braincomms/fcad225)
Supplement: fcad225_Supplementary_Data [file fcad225_supplementary_data.doc]

# Supplementary Material

**Supplementary Table 1. An overview of the analytical approach to covariates, interactions, multiple testing correction and sensitivity analyses for each domain investigated***. FDR=false discovery rate. N/A=not applicable. WMHV=white matter hyperintensity volume.*

|  | **Concurrent pathology** | **Concurrent cognition** | **Demographics** | **Cardiovascular health** |
| --- | --- | --- | --- | --- |
| Covariate approach | Sex, age and mutual adjustment for pathology | Sex, age and mutual adjustment for sub-tests | Mutual adjustment for all demographics including sex and age. | Sex, age |
| Test for Sex interaction | Yes | Yes | N/A (primary model includes sex) | Yes |
| FDR-corrected | Yes | Yes | Yes | Yes |
| Sensitivity – adjust for WMHV | N/A (primary model includes WMHV) | Yes | Yes | Yes |
| Sensitivity – adjust for APOE-e4 status | Yes | Yes | N/A (primary model includes APOE) | Yes |

**Supplementary Table 2. Associations between concurrent imaging correlates with standardized global mean normal appearing white matter (NAWM) parameters of fractional anisotropy (FA); neurite density index (NDI); mean diffusivity (MD) and orientation dispersion index (ODI) at age 69-71 years.**

| Concurrent Pathology | | | | |
| --- | --- | --- | --- | --- |
|  | **β** | **FDR-corrected**  **p value** | **95% CI** | **Sex**  **interaction** |
| **FA** |  |  |  |  |
| Whole brain volume | -0.02 | 0.05 | -0.05-0.00 | 0.19 |
| log(WMHV) | -0.09 | **<0.01** | -0.11--0.06 | 0.46 |
| Amyloid SUVR | -0.03 | **0.04** | -0.04--0.01 | **0.01** |
| Amyloid: men | -0.04 | **0.03** | -0.08--0.01 |  |
| Amyloid: women | 0.01 | 0.54 | -0.02-0.05 |  |
| **MD** |  |  |  |  |
| Whole brain volume | 0.01 | 0.50 | -0.02-0.05 | 0.29 |
| log(WMHV) | 0.14 | **<0.01** | 0.10-0.17 | 0.57 |
| Amyloid SUVR | 0.06 | **0.04** | 0.01-0.11 | **0.01** |
| Amyloid: men | 0.06 | **0.02** | 0.01-0.11 |  |
| Amyloid: women | -0.03 | 0.31 | -0.07-0.02 |  |
| **NDI** |  |  |  |  |
| Whole brain volume | 0.04 | 0.13 | -0.01-0.09 | 0.55 |
| log(WMHV) | -0.17 | **<0.01** | -0.22--0.12 | 0.30 |
| Amyloid SUVR | -0.05 | 0.13 | -0.13-0.01 | **0.02** |
| Amyloid: men | -0.07 | 0.05 | -0.14-0.01 |  |
| Amyloid: women | 0.05 | 0.14 | -0.02-0.12 |  |
| **ODI** |  |  |  |  |
| Whole brain volume | 0.01 | 0.33 | -0.01-0.02 | 0.31 |
| log(WMHV) | 0.00 | 0.98 | -0.01-0.01 | 0.11 |
| Amyloid SUVR | 0.00 | 0.30 | -0.02-0.01 | 0.22 |
| Amyloid: men | -0.01 | 0.36 | -0.03-0.01 |  |
| Amyloid: women | 0.01 | 0.46 | -0.01-0.03 |  |

Regression coefficients and 95% confidence intervals (CI) of the standardized estimates reflect the differences in mean of the standardized NAWM outcome by one standard deviation change in the predictor variable. All concurrent imaging correlates were assessed in the same model and adjusted for sex, age at scan and APOE-ε4 status, per NAWM metric. A sex and amyloid SUVR significant interaction emerged, so amyloid SUVR results were stratified by sex. P-values that pass false discovery rate (FDR) correction are highlighted in bold. *WMHV=White matter hyperintensity volume; SUVR= Standardised uptake value ratio.*

**Supplementary Table 3. Associations between concurrent cognition correlates (preclinical Alzheimer’s cognitive composite (PACC) and its four sub-components) with standardized global mean normal appearing white matter (NAWM) parameters of fractional anisotropy (FA); neurite density index (NDI); mean diffusivity (MD) and orientation dispersion index (ODI) at age 69-71 years.**

| Concurrent cognition | | | | |
| --- | --- | --- | --- | --- |
|  | **β** | **FDR corrected**  **p-value** | **95% CI** | **Sex interaction** |
| **FA** |  |  |  |  |
| PACC total score | 0.01 | 0.64 | -0.02-0.03 | 0.98 |
| Digit symbol | 0.01 | 0.36 | -0.01-0.04 | 0.89 |
| Logical memory | 0.00 | 0.90 | -0.03-0.03 | 0.42 |
| FNAME score | 0.00 | 0.91 | -0.03-0.03 | 0.61 |
| MMSE score | -0.01 | 0.59 | -0.04-0.02 | 0.45 |
| **MD** |  |  |  |  |
| PACC total score | 0.00 | 0.94 | -0.04-0.04 | 0.52 |
| Digit symbol | -0.02 | 0.29 | -0.06-0.02 | 0.61 |
| Logical memory | 0.01 | 0.64 | -0.03-0.05 | 0.14 |
| FNAME score | 0.00 | 0.98 | -0.04-0.04 | 0.23 |
| MMSE score | 0.01 | 0.68 | -0.03-0.05 | 0.30 |
| **NDI** |  |  |  |  |
| PACC total score | 0.04 | 0.15 | -0.01-0.09 | 0.26 |
| Digit symbol | 0.07 | 0.02 | 0.01-0.12 | 0.26 |
| Logical memory | -0.02 | 0.46 | -0.08-0.04 | 0.90 |
| FNAME score | 0.01 | 0.80 | -0.05-0.07 | 0.95 |
| MMSE score | 0.01 | 0.82 | -0.05-0.06 | 0.97 |
| **ODI** |  |  |  |  |
| PACC total score | -0.01 | 0.21 | -0.02-0.01 | 0.59 |
| Digit symbol | 0.00 | 0.66 | -0.01-0.02 | 0.85 |
| Logical memory | -0.01 | 0.09 | -0.03-0.00 | 0.44 |
| FNAME score | 0.00 | 0.59 | -0.02-0.01 | 0.49 |
| MMSE score | 0.00 | 0.60 | -0.01-0.02 | 0.96 |

Regression coefficients and 95% confidence intervals (CI) of the standardized estimates reflect the differences in mean of the standardized NAWM outcome by one standard deviation change in the predictor variable. There was no evidence of sex interactions so all models are adjusted for sex, age at scan and APOE-ε4 status, per NAWM metric. All sub-components of the PACC were assessed in the same model, per NAWM metric*.* P-values that pass false discovery rate (FDR) correction are highlighted in bold. *PACC=Preclinical Alzheimer Cognitive Composite; MMSE=mini mental state examination.*

**Supplementary Table 4:** **Associations between life course demographics with mean normal appearing white matter (NAWM) parameters of fractional anisotropy (FA); neurite density index (NDI); mean diffusivity (MD); orientation dispersion index (ODI) at age 69-71.**

| Demographics | | | | |
| --- | --- | --- | --- | --- |
|  |  | **β** | **FDR p-value** | **95% CI** |
| **FA** |  |  |  |  |
| Sex |  | -0.11 | **<0.01** | -0.17--0.06 |
| Age |  | -0.04 | **0.03** | -0.08--0.00 |
| Childhood cognition |  | 0.00 | 0.75 | -0.03-0.03 |
| Education | No qualifications | Reference | . | 0.00-0.00 |
|  | up to 16 | 0.00 | 0.94 | -0.08-0.09 |
|  | 17+ | 0.03 | 0.51 | -0.06-0.12 |
| Social class | Non-manual | Reference | . | 0.00-0.00 |
|  | manual | 0.02 | 0.59 | -0.04-0.07 |
| APOE-e4 status | No alleles | Reference | . | 0.00-0.00 |
|  | 1 or 2 e4 alleles | 0.00 | 0.89 | -0.05-0.06 |
| **MD** |  |  |  |  |
| Sex |  | 0.07 | 0.07 | -0.01-0.15 |
| Age |  | 0.07 | **0.01** | 0.01-0.13 |
| Childhood cognition |  | 0.01 | 0.52 | -0.03-0.06 |
| Education | No qualifications | Reference | . | 0.00-0.00 |
|  | up to 16 | -0.02 | 0.79 | -0.14-0.10 |
|  | 17+ | -0.02 | 0.71 | -0.15-0.10 |
| Social class | Non-manual | Reference | . | 0.00-0.00 |
|  | manual | 0.00 | 0.92 | -0.08-0.08 |
| APOE status | No alleles | Reference | . | 0.00-0.00 |
|  | 1 or 2 e4 alleles | 0.01 | 0.74 | -0.07-0.10 |
| **NDI** |  |  |  |  |
| Sex |  | -0.1 | 0.09 | -0.21-0.02 |
| Age |  | -0.09 | **0.02** | -0.18--0.01 |
| Childhood cognition |  | 0.02 | 0.57 | -0.04-0.08 |
| Education | No qualifications | Reference | . | 0.00-0.00 |
|  | up to 16 | -0.06 | 0.48 | -0.24-0.11 |
|  | 17+ | -0.05 | 0.57 | -0.23-0.13 |
| Social class | Non-manual | Reference | . | 0.00-0.00 |
|  | manual | -0.02 | 0.75 | -0.14-0.10 |
| APOE status | No alleles | Reference | . | 0.00-0.00 |
|  | 1 or 2 e4 alleles | 0.04 | 0.53 | -0.08-0.16 |
| **ODI** |  |  |  |  |
| Sex |  | 0.06 | **<0.01** | 0.03-0.09 |
| Age |  | 0.00 | 0.69 | -0.02-0.03 |
| Childhood cognition |  | 0.00 | 0.67 | -0.02-0.01 |
| Education | No qualifications | Reference | . | 0.00-0.00 |
|  | up to 16 | 0.00 | 0.87 | -0.04-0.05 |
|  | 17+ | -0.03 | 0.28 | -0.07-0.02 |
| Social class | Non-manual | Reference | . | 0.00-0.00 |
|  | manual | -0.01 | 0.44 | -0.04-0.02 |
| APOE status | No alleles | Reference | . | 0.00-0.00 |
|  | 1 or 2 e4 alleles | -0.01 | 0.56 | -0.04-0.02 |

Regression coefficients and 95% confidence intervals (CI) of the standardized estimates reflect the differences in mean of the standardized NAWM outcome by one standard deviation change in the predictor variable. Estimates for age reflect the differences in mean associated with a 1-year increase in age and for an increase of 1 standard deviation for childhood cognition. The predictors were assessed in the same model to estimate the independence of effects, per NAWM metric. P-values that pass false discovery rate (FDR) correction are highlighted in bold. *SEP=socioeconomic position.*

**Supplementary Table 5:** **Associations between life course vascular risk and blood pressure with mean normal appearing white matter (NAWM) parameters of fractional anisotropy (FA); neurite density index (NDI); mean diffusivity (MD); orientation dispersion index (ODI) at age 69-71.**

| **FA** | **β** | **FDR p-value** | **95% CI** | **Sex interaction** |
| --- | --- | --- | --- | --- |
| Framingham-Heart-Study Cardiovascular Risk Score (FHS-CVS)%: | | | | |
| at age 36 years | -0.01 | 0.66 | -0.05-0.03 | 0.53 |
| at age 53 years | -0.03 | 0.06 | -0.06-0.00 | **<0.01** |
| at age 69 years | -0.06 | **<0.01** | -0.09--0.02 | 0.05 |
| Systolic blood pressure (SBP): | | | | |
| at age 36 years | 0.00 | 0.99 | -0.03-0.03 | 0.79 |
| at age 43 years | 0.01 | 0.46 | -0.02-0.04 | 0.60 |
| at age 53 years | -0.03 | 0.04 | -0.05--0.00 | **0.01** |
| at age 60-64 years | 0.00 | 0.84 | -0.03-0.02 | 0.28 |
| at age 69 years | -0.02 | 0.15 | -0.05-0.01 | 0.80 |
| Diastolic blood pressure (DBP): | | | | |
| at age 36 years | 0.00 | 0.94 | -0.03-0.03 | 0.88 |
| at age 43 years | 0.00 | 0.78 | -0.03-0.02 | 0.27 |
| at age 53 years | -0.04 | **0.01** | -0.07--0.01 | **0.01** |
| at age 60-64 years | -0.03 | 0.03 | -0.06--0.00 | **0.01** |
| at age 69 years | -0.02 | 0.15 | -0.05-0.01 | 0.80 |
| Systolic blood pressure change (SBP): | | | | |
| 36 - 43 | 0.01 | 0.42 | -0.02-0.04 | 0.55 |
| 43 - 53 | -0.03 | 0.06 | -0.06-0.00 | **0.02** |
| 53 -60 | 0.02 | 0.28 | -0.01-0.04 | 0.61 |
| 60- 69 | -0.02 | 0.13 | -0.05-0.01 | 0.38 |
| Diastolic blood pressure change (DBP): | | | | |
| 36 - 43 | -0.01 | 0.39 | -0.04-0.02 | 0.08 |
| 43 - 53 | -0.03 | **0.03** | -0.06--0.00 | **0.04** |
| 53 -60 | -0.01 | 0.54 | -0.04-0.02 | 0.52 |
| 60- 69 | -0.01 | 0.66 | -0.04-0.02 | 0.41 |
| BMI at age 70 years | -0.01 | 0.32 | -0.04-0.01 | 0.21 |

Regression coefficients and 95% confidence intervals (CI) of the standardized estimates reflect the differences in mean of the standardized NAWM outcome by one standard deviation change in the predictor variable. *FHS=Framingham-Heart-Study Cardiovascular Risk Score; SBP=systolic blood pressure; DBP=diastolic blood pressure.* P-values that pass false discovery rate (FDR) correction are highlighted in bold.

| MD | **β** | **FDR p-value** | **95% CI** | **Sex interaction** |
| --- | --- | --- | --- | --- |
| Framingham-Heart-Study Cardiovascular Risk Score (FHS-CVS)%: | | | | |
| at age 36 years | 0.01 | 0.74 | -0.04-0.06 | 0.59 |
| at age 53 years | 0.05 | 0.04 | 0.00-0.09 | **0.02** |
| at age 69 years | 0.09 | **<0.01** | 0.04-0.14 | 0.12 |
| Systolic blood pressure (SBP): | | | | |
| at age 36 years | 0.00 | 0.85 | -0.04-0.05 | 0.91 |
| at age 43 years | 0.00 | 0.97 | -0.04-0.04 | 0.49 |
| at age 53 years | 0.04 | **0.03** | 0.00-0.08 | **0.02** |
| at age 60-64 years | 0.01 | 0.44 | -0.02-0.05 | 0.29 |
| at age 69 years | 0.03 | 0.06 | -0.00-0.07 | 0.64 |
| Diastolic blood pressure (DBP): | | | | |
| at age 36 years | 0.00 | 1.00 | -0.04-0.04 | 0.89 |
| at age 43 years | 0.01 | 0.48 | -0.03-0.05 | 0.29 |
| at age 53 years | 0.05 | **0.02** | 0.01-0.09 | **0.04** |
| at age 60-64 years | 0.03 | 0.08 | -0.00-0.07 | 0.07 |
| at age 69 years | 0.03 | 0.06 | -0.00-0.07 | 0.64 |
| Systolic blood pressure change (SBP): | | | | |
| 36 - 43 | 0.00 | 0.93 | -0.04-0.04 | 0.29 |
| 43 - 53 | 0.04 | 0.04 | 0.00-0.08 | 0.11 |
| 53 -60 | -0.01 | 0.57 | -0.05-0.03 | 0.63 |
| 60- 69 | 0.03 | 0.09 | -0.01-0.08 | 0.44 |
| Diastolic blood pressure change (DBP): | | | | |
| 36 - 43 | 0.02 | 0.25 | -0.02-0.06 | 0.10 |
| 43 - 53 | 0.04 | 0.04 | 0.00-0.08 | 0.17 |
| 53 -60 | 0.01 | 0.76 | -0.03-0.05 | 0.91 |
| 60- 69 | 0.02 | 0.30 | -0.02-0.06 | 0.96 |
| BMI at age 70 years | 0.01 | 0.51 | -0.02-0.05 | 0.13 |

Regression coefficients and 95% confidence intervals (CI) of the standardized estimates reflect the differences in mean of the standardized NAWM outcome by one standard deviation change in the predictor variable. *FHS=Framingham-Heart-Study Cardiovascular Risk Score; SBP=systolic blood pressure; DBP=diastolic blood pressure.* P-values that pass false discovery rate (FDR) correction are highlighted in bold.

| NDI | **β** | **FDR p-value** | **95% CI** | **Sex interaction** |
| --- | --- | --- | --- | --- |
| Framingham-Heart-Study Cardiovascular Risk Score (FHS-CVS)%: | | | | |
| at age 36 years | -0.01 | 0.87 | -0.08-0.07 | 0.78 |
| at age 53 years | -0.10 | **<0.01** | -0.17--0.03 | **0.00** |
| at age 69 years | -0.10 | **<0.01** | -0.17--0.03 | **0.02** |
| Systolic blood pressure (SBP): | | | | |
| at age 36 years | 0.01 | 0.87 | -0.06-0.07 | 0.94 |
| at age 43 years | -0.02 | 0.57 | -0.07-0.04 | 0.27 |
| at age 53 years | -0.07 | **0.01** | -0.12--0.01 | **0.00** |
| at age 60-64 years | 0.00 | 0.99 | -0.05-0.05 | 0.66 |
| at age 69 years | -0.02 | 0.37 | -0.08-0.03 | 0.62 |
| Diastolic blood pressure (DBP): | | | | |
| at age 36 years | 0.03 | 0.38 | -0.03-0.08 | 0.59 |
| at age 43 years | -0.01 | 0.81 | -0.06-0.05 | 0.28 |
| at age 53 years | -0.04 | 0.13 | -0.10-0.01 | **0.01** |
| at age 60-64 years | -0.04 | 0.09 | -0.10-0.01 | **0.04** |
| at age 69 years | -0.02 | 0.37 | -0.08-0.03 | 0.62 |
| Systolic blood pressure change (SBP): | | | | |
| 36 - 43 | -0.01 | 0.71 | -0.07-0.05 | 0.35 |
| 43 - 53 | -0.06 | **0.02** | -0.12--0.01 | **<0.01** |
| 53 -60 | 0.05 | 0.11 | -0.01-0.10 | 0.13 |
| 60- 69 | -0.02 | 0.60 | -0.07-0.04 | 0.76 |
| Diastolic blood pressure change (DBP): | | | | |
| 36 - 43 | -0.02 | 0.40 | -0.08-0.03 | 0.10 |
| 43 - 53 | -0.04 | 0.17 | -0.10-0.02 | 0.06 |
| 53 -60 | -0.02 | 0.54 | -0.07-0.04 | 0.83 |
| 60- 69 | -0.01 | 0.86 | -0.06-0.05 | 0.75 |
| BMI at age 70 years | -0.03 | 0.18 | -0.09-0.02 | 0.20 |

Regression coefficients and 95% confidence intervals (CI) of the standardized estimates reflect the differences in mean of the standardized NAWM outcome by one standard deviation change in the predictor variable. *FHS=Framingham-Heart-Study Cardiovascular Risk Score; SBP=systolic blood pressure; DBP=diastolic blood pressure.* P-values that pass false discovery rate (FDR) correction are highlighted in bold.

| ODI | **β** | **FDR p-value** | **95% CI** | **Sex interaction** |
| --- | --- | --- | --- | --- |
| Framingham-Heart-Study Cardiovascular Risk Score (FHS-CVS)%: | | | | |
| at age 36 years | 0.01 | 0.36 | -0.01-0.03 | 0.45 |
| at age 53 years | -0.01 | 0.48 | -0.03-0.01 | 0.72 |
| at age 69 years | 0.00 | 0.62 | -0.01-0.02 | 0.67 |
| Systolic blood pressure (SBP): | | | | |
| at age 36 years | 0.00 | 0.94 | -0.02-0.02 | 0.46 |
| at age 43 years | -0.01 | 0.13 | -0.03-0.00 | 0.49 |
| at age 53 years | 0.00 | 0.82 | -0.02-0.01 | 0.40 |
| at age 60-64 years | 0.00 | 0.81 | -0.02-0.01 | 0.71 |
| at age 69 years | 0.00 | 0.70 | -0.02-0.01 | 0.39 |
| Diastolic blood pressure (DBP): | | | | |
| at age 36 years | 0.00 | 0.82 | -0.01-0.02 | 0.85 |
| at age 43 years | 0.00 | 0.68 | -0.02-0.01 | 0.71 |
| at age 53 years | 0.01 | 0.23 | -0.01-0.02 | 0.40 |
| at age 60-64 years | 0.01 | 0.30 | -0.01-0.02 | 0.21 |
| at age 69 years | 0.00 | 0.70 | -0.02-0.01 | 0.39 |
| Systolic blood pressure change (SBP): | | | | |
| 36 - 43 | -0.01 | 0.08 | -0.03-0.00 | 0.30 |
| 43 - 53 | -0.01 | 0.45 | -0.02-0.01 | 0.51 |
| 53 -60 | 0.00 | 0.86 | -0.02-0.01 | 0.56 |
| 60- 69 | 0.00 | 0.91 | -0.02-0.01 | 0.85 |
| Diastolic blood pressure change (DBP): | | | | |
| 36 - 43 | 0.00 | 0.86 | -0.02-0.01 | 0.99 |
| 43 - 53 | 0.00 | 0.64 | -0.01-0.02 | 0.45 |
| 53 -60 | 0.00 | 0.55 | -0.01-0.02 | 0.24 |
| 60- 69 | -0.01 | 0.30 | -0.02-0.01 | 0.06 |
| BMI at age 70 years | 0.01 | 0.07 | -0.00-0.03 | 0.21 |

Regression coefficients and 95% confidence intervals (CI) of the standardized estimates reflect the differences in mean of the standardized NAWM outcome by one standard deviation change in the predictor variable. *FHS=Framingham-Heart-Study Cardiovascular Risk Score; SBP=systolic blood pressure; DBP=diastolic blood pressure.* P-values that pass false discovery rate (FDR) correction are highlighted in bold.

**Supplementary Fig *1*: Associations between concurrent imaging correlates with standardized global mean normal appearing white matter (NAWM) parameters of fractional anisotropy (FA); neurite density index (NDI); mean diffusivity (MD) and orientation dispersion index (ODI) at age 69-71 years.** Regression coefficient plot of the standardized estimates which reflect the differences in mean of the standardized NAWM outcome by one standard deviation change in the predictor variable. Lines indicate the widths of the 95% confidence intervals. All concurrent imaging correlates were assessed in the same model and adjusted for sex and age at scan and APOE-e4 status, per NAWM metric. A sex and amyloid SUVR significant interaction emerged, so amyloid SUVR results were stratified by sex. Associations that survived false discovery rate correction are indicated by an asterisk. *WMHV=White matter hyperintensity volume; SUVR= Standardised uptake value ratio; TIV=Total intracranial volume.*

**Supplementary Fig *2*: Associations between life course demographics with mean normal appearing white matter (NAWM) parameters of fractional anisotropy (FA); neurite density index (NDI); mean diffusivity (MD); orientation dispersion index (ODI) at age 69-71.** Regression coefficients and 95% confidence intervals (CI) of the standardized estimates reflect the differences in mean of the standardized NAWM outcome by one standard deviation change in the predictor variable. Estimates for age reflect the differences in mean associated with a 1-year increase in age and for an increase of 1 standard deviation for childhood cognition. Lines indicate the widths of the 95% confidence intervals. The predictors were assessed in the same model to estimate the independence of effects, per NAWM metric, with additional adjustment from white matter hyperintensity volume. Associations that survived false discovery rate correction are indicated by an asterisk. *SEP=socioeconomic position.*

**Supplementary Fig 3: Life course vascular risk and blood pressure associations with mean normal appearing white matter (NAWM) parameters of fractional anisotropy (FA); neurite density index (NDI); mean diffusivity (MD); orientation dispersion index (ODI) at age 69-71.** Regression coefficient plot of the standardized estimates which reflect the change (differences in mean) of the standardized NAWM outcome by one standard deviation change in the predictor variable. Lines indicate the widths of the 95% confidence intervals. FHS=Framingham-Heart-Study Cardiovascular Risk Score; SBP=systolic blood pressure; DBP=diastolic blood pressure. Associations that survived false discovery rate correction are indicated by an asterisk. All models were run individually per NAWM metric, and are adjusted for sex and age at scan and white matter hyperintensity volume.

**Supplementary Fig 4: Life course vascular risk and blood pressure associations with mean normal appearing white matter (NAWM) parameters of fractional anisotropy (FA); neurite density index (NDI); mean diffusivity (MD); orientation dispersion index (ODI) at age 69-71.** Regression coefficient plot of the standardized estimates which reflect the change (differences in mean) of the standardized NAWM outcome by one standard deviation change in the predictor variable. Lines indicate the widths of the 95% confidence intervals. FHS=Framingham-Heart-Study Cardiovascular Risk Score; SBP=systolic blood pressure; DBP=diastolic blood pressure. Associations that survived false discovery rate correction are indicated by an asterisk. All models were run individually per NAWM metric, and are adjusted for sex and age at scan and APOE-e4 status.

**Supplementary Fig 5: Sex differences in vascular risk and blood pressure associations at age 53 with mean normal appearing white matter (NAWM) parameters of fractional anisotropy (FA); neurite density index (NDI); mean diffusivity (MD); orientation dispersion index (ODI) at age 69-71.** Regression coefficient plot of the standardized estimates which reflect the change (differences in mean) of the standardized NAWM outcome by one standard deviation change in the predictor variable. Lines indicate the widths of the 95% confidence intervals. Associations that survived false discovery rate correction are indicated by an asterisk.  *FHS=Framingham-Heart-Study Cardiovascular Risk Score; SBP=systolic blood pressure; DBP=diastolic blood pressure.* All models were run individually per NAWM metric, and are adjusted for sex and age at scan and white matter hyperintensity volume.

**Supplementary Fig 6: Sex differences in vascular risk and blood pressure associations at age 53 with mean normal appearing white matter (NAWM) parameters of fractional anisotropy (FA); neurite density index (NDI); mean diffusivity (MD); orientation dispersion index (ODI) at age 69-71.** Regression coefficient plot of the standardized estimates which reflect the change (differences in mean) of the standardized NAWM outcome by one standard deviation change in the predictor variable. Lines indicate the widths of the 95% confidence intervals. Associations that survived false discovery rate correction are indicated by an asterisk.  *FHS=Framingham-Heart-Study Cardiovascular Risk Score; SBP=systolic blood pressure; DBP=diastolic blood pressure.* All models were run individually per NAWM metric, and are adjusted for sex and age at scan and APOE-e4 status.
